# Transmission of oral microbiota to the biliary tract during endoscopic retrograde cholangiography

**DOI:** 10.1186/s12876-023-02721-7

**Published:** 2023-04-03

**Authors:** Maria Effenberger, Ramona Al-Zoairy, Ronald Gstir, Ivo Graziadei, Hubert Schwaighofer, Herbert Tilg, Heinz Zoller

**Affiliations:** 1 Department of Internal Medicine I, Gastroenterology, Hepatology, Endocrinology and Metabolism, Medical University of Innsbruck, Anichstrasse 35, Innsbruck, 6020 Austria; 2 Division of Hygiene and Medical Microbiology, Medical University of Innsbruck, Innsbruck, Austria; 3 Department of Internal Medicine, Academic Teaching Hospital Hall, Hall/Tirol, Austria; 4 Christian Doppler Laboratory on Iron and Phosphate Biology, Medical University of Innsbruck, Innsbruck, Austria

**Keywords:** ERC, Oral microbiome, Cholangitis, Biliary tract

## Abstract

**Background:**

Endoscopic retrograde cholangiography (ERC) possesses a translocation risk of microbes to the biliary system. We studied bile contamination during ERC and its impact on patients’ outcome in a real-life-situation.

**Methods:**

Ninety-nine ERCs were analyzed and microbial samples were taken from the throat before and from bile during ERC and from irrigation fluid of the duodenoscope before and after ERC.

**Results:**

91.2% of cholangitis patients had detectable microbes in the bile (sensitivity 91%), but the same was true for 86.2% in the non-cholangitis group. *Bacteroides fragilis* (*p*=0.015) was significantly associated with cholangitis. In 41.7% of ERCs with contaminated endoscopes these microbes were found in the bile after the procedure. Analysis of duodenoscopes’ irrigation liquid after ERC matched the microbial bile analysis of these patients in 78.8%. Identical microbial species were in throat and in bile samples of the same ERC in 33% of all cases and in 45% in the non-cholangitis group. Transmission of microbes to the biliary tract did not result in more frequent cholangitis, longer hospital stays, or worse outcome.

**Conclusions:**

During ERC bile samples are regularly contaminated with microbes of the oral cavity but it did not affect clinical outcome.

**Supplementary Information:**

The online version contains supplementary material available at 10.1186/s12876-023-02721-7.

## Background

Distal biliary strictures (DBS) are common and may be caused by both malignant and benign pathologies [1]. Cholangitis is a frequent and potentially serious complication in patients with bile duct obstruction. Biliary decompression, one of the key elements of DBS and cholangitis treatment, is most often achieved by techniques applied at endoscopic retrograde cholangiography (ERC). Multiple studies support superior outcomes and decreased mortality rates with ERC compared to interventional radiology or surgical modalities [2–4].

At present, these highly beneficial ERC procedures are primarily performed using reusable duodenoscopes, but due to their complex architecture and design, duodenoscopes are difficult to clean [5, 6]. Insufficient cleaning results in remaining microbiological debris in patient-ready duodenoscopes, which might cause patient-to-patient cross-contamination and subsequent infections [7, 8]. Thus, there are controversies with regards to the impact of contaminated duodenoscopes, and whether such equipment can cause post-endoscopic device-related infections that could negatively affect patient safety [8].

ERC was always assumed to possess an inherent translocation risk of microbial species between the oral cavity and the biliary system. Additionally, endoscopic manipulations such as sphincterotomy or biliary stent insertion during ERC may increase the risk for translocation of microbes from the upper gastrointestinal tract into the bile by disrupting anatomical and functional barriers. Therefore, one complication of ERC that occurs in up to 0.5-3.0% is cholangitis, which can cause life threatening septicemia [9, 10].

The aim of the present study was to assess biliary contamination during the endoscopic procedure and consequently the validity of microbiological test results obtained from bile samples during ERCs. Therefore, known risk factors for cholangitis, biochemical indicators of cholangitis, microbiological samples from duodenoscopes before and after ERC, bile samples, and throat swabs were taken before and during ERC. Data were analyzed regarding the clinical diagnosis of cholangitis as defined by the Tokyo guidelines of 2018 [11].

## Methods

### Sample collection

This retrospective study comprises data from the hygiene surveillance program and includes 99 ERCs in the University Hospital of the Medical University of Innsbruck between November 2010 and October 2011. None of the patients received antibiotic therapy within 3 months before hospitalization for ERC. In 40 ERCs antibiotic therapy was administered within 24 hours before or during ERC. The antibiotic therapy included quinolones (*n*=16), β-Lactam antibiotics/β-Lactamase inhibitors (*n*=13), nitroimidazoles (*n*=8), or 3^rd^ generation cephalosporines (*n*=3).

Each examination included two microbial samples coming from a throat swab taken before endoscopy and a bile sample taken during the examination. Additionally two microbial samples were taken from the duodenoscope: one before and one after ERC. For bile collection a bile specimen was collected after cannulation of the papilla by passing a sterile 5 French standard ERC- or balloon-catheter into the common bile duct and aspirating bile into a sterile 10 mL syringe (Injekt, B. Braun, Melsungen, Germany). 20 ml sterile-deionized water (B. Braun, Melsungen, Germany) were flushed through the duodenoscope immediately before and after ERC. Aqua bidest was collected into a sterile 20 mL syringe (Injekt, B. Braun, Melsungen, Germany).

### Microbiological analysis and susceptibility testing

Bile aspirates and irrigation fluid were transported in sterile test tubes, aerobic or anaerobic culture bottles and throat swabs were transported in a sterile test tubes. Samples reached the laboratory within one hour for immediate processing. Bile samples and irrigation liquid of duodenoscopes were cultivated on Columbia blood agar, Bacteroides Bile Esculin (BBE) and Schaedler anaerobic agar (all Becton Dickinson, Heidelberg, Germany) and incubated for 48 h at 37°C under aerobic (for Columbia blood agar) or anaerobic conditions. Throat swabs were cultivated on Columbia blood agar, boiled blood agar, and Max Conkey agar. Microbial identification was done by matrix-assisted laser desorption ionization time-of-flight mass spectrometry (MALDI-TOF, Bruker, Bremen, Germany) using the direct smear method for samples that were taken in the year 2011. A score above 1.7 was considered valid. Prior to 2011 biochemical identification using standard microbiological procedures such as API- or VITEK-system (Biomerieux, Marcy-l´Etoile, France) was conducted. Negative cultures were kept for a total of 5 days before they were discarded and classified as sterile.

Microorganisms were categorized into two groups:

- Likely pathogenic microorganisms (group 1): considered as causative pathogen if identified in bile aspirates of patients with cholangitis (e.g. Enterobacteriaceae, *Pseudomonas aeruginosa*, *Bacteroides fragilis*,* Enterococcus faecalis* and *faecium, Staphylococcus aureus, Streptococcus anginosus* and *milleri, Candida spp*.)
- Facultative or unusual pathogens (group 2): microorganisms that rarely cause cholangitis (e.g. *Streptococcus viridans*, coagulase-negative staphylococci*, Enterococcus avium*, *gallinarum* and *casseliflavus, Bacillus spp.*). Contamination or transient colonization cannot be excluded in these cases.

Antibiotic susceptibility testing was performed according to the CLSI guidelines [12] using the VITEK system (Biomerieux, Marcy-l´Etoile, France).

### Clinical data

Clinical Data were extracted from the local health information system (Cerner, North Kansas City, MO) and the local medical information software (Fujifilm, Tokyo, Japan).

### Diagnosis of acute cholangitis

Demographic, clinical and biochemical parameters were recorded to assess each patient for the presence of acute cholangitis according to the “Tokyo Guidelines 2018” [11] at the time of ERC.

### Endoscopy equipment

Duodenoscopes were from Fujinon (Fujinon Europe, Ltd., Duesseldorf, Germany). Bile was aspirated with bile cannulas (Contour Cannula, Boston Scientific, Natick, MA). In such cases where sphincterotomy was performed, an ultratome (Triple Lumen Sphincterotome, Boston Scientific, Natick, MA) was used. Dilation of stenosis was performed with dilating balloons (Max Force Biliary Balloon dilation, Boston Scientific, Natick, MA).

### Double-reprocessing High-Level Disinfection (DHLD) protocol for cleaning duodenoscopes

After each ERC, the duodenoscope’s external surface was wiped and the channels were irrigated with Neodisher EndoClean 10% solution (Dr. Weigert UK Ltd, London, UK). After precleaning, the duodenoscope was immersed in this enzymatic detergent solution and thoroughly cleaned using a single-use, manufacturer recommended brush to remove visible debris from all areas of the duodenoscope (elevator, -recess, -locking mechanism, suction-, air/water-, and instrument-channel port). Manual cleaning was continued until the surface was free of apparent debris. The instrument channels and the suction port were irrigated with the detergent solution and brushed with special brushes (Fujifilm, Tokyo, Japan). Brushes were also used for cleaning of the elevator. Following manual cleaning, the duodenoscope was placed in an automated endoscope reprocessor (Belimed WD430, Zug, Switzerland) for a total cycle time of approximately 60 minutes, using the high-level disinfectant Neodisher EndoSept GA and the high-level detergent Neodisher EndoClean (both Dr. Weigert UK Ltd, London, UK). The entire process of manual cleaning and reprocessing was then repeated to complete the DHLD protocol. All duodenoscopes were dried thoroughly and were stored in ambient air, except those selected for surveillance cultures. There was no forced air drying and duodenoscopes not in use for 7 days underwent repeated HLD. Cleaning and HLD was performed by specially trained staff.

Routine bacteriological surveillance was performed every 3 months by the infection control team of the Division of Hygiene and Medical Microbiology, Medical University of Innsbruck. It included examination of swabs from the working channels and the functionality of the washing machine to sterilize standardized bacterial cultures.

### Statistical analysis

Statistical analysis was performed using the chi-square test and association of variables was assessed by Pearson's correlation. A *P* value < 0.05 was considered statistically significant. The diagnostic properties of microbiological examination of bile aspirates during ERC for the diagnosis of cholangitis were calculated using descriptive statistics. Diagnostic test performance was calculated from the contingency table as indicated in the results section.

In a first step, univariate Cox proportional hazards regression was calculated to identify risk factors predictive for cholangitis in total. In the second step, the subgroups in the univariate analysis were included in a multivariate Cox regression model with stepwise backwards selection. Kaplan-Meier estimates were created and compared using log-rank test and chi-square. Statistical analysis was carried out in IBM SPSS Statistics (IBM, version 24.0, New York City, NY, USA) and GraphPad PRISM 5 (La Jolla, CA).

### Ethical consideration

Informed consent was obtained from each patient included in the study. The study protocol conforms to the ethical guidelines of the 1975 Declaration of Helsinki (6th revision, 2008) as reflected in a priori approval by the institution's human research committee. The study protocol was approved by the institutional ethics commission (1100/2022).

## Results

### Positive microbial bile culture has high sensitivity for cholangitis diagnosis

To assess the microbial composition of bile aspirates during ERC, data from a total of 99 ERCs in 49 patients were included during the study period. Characteristics of these in-hospital patients with indication for ERC, underlying aetiologies, and co-morbidities are listed in Tables 1 and 2. Twenty-nine had one ERC and the remaining 20 patients required multiple ERCs with an actual number of endoscopies ranging from 2 to 7. In 28 patients, endoscopic papillotomy (EPT) was performed and 21 patients underwent previous ERCs with EPT (Tables 1 and 2).

**Table 1:** Patients´ characteristics and characteristics of ERCs

	**Total**	**Cholangitis**	**No Cholangitis**	***p*-value**
**Number of patients (%)**	49 (100%)	16 (32.7%)	33 (67.4%)	–-
**Number of female patients**	11 (21.4%)	4 (7.4%)	7 (14.4%)	0.394
**Age (years)**	61.4 ± 11.6	62.3 ± 12.8	60.7 ± 8.4	0.341
**Smoker**	18 (36.7%)	6 (37.5%)	12 (36.3%)	0.519
**EPT before inclusion**	21 (42.9%)	9 (56.1%)	12 (36.4%)	0.134
**EPT during the study period**	28 (57.1%)	7 (43.2%)	21 (63.6%)	0.213
**Diabetes mellitus**	8 (16.3%)	3 (18.8%)	5 (15.2%)	0.732
**Malignant disease**	10 (20.4%)	4 (25%)	6 (18.2%)	0.659
**Malignant disease causing bile duct stenosis**	7 (14,3%)	2 (12.5%)	5 (15.2%)	0.823
**OLT prior to study inclusion**	17 (34.7%)	4 (25%)	13 (39.4%)	0.501
**CRP (mg/dl)**	0.95 (0.4–11.3)	6.2 (3.4–11.3)	0.6 (0.4–1)	**0.011**
**AST (U/l)**	61 (39–102)	68 (43.8–133.5)	57 (37–80.5)	0.240
**ALT (U/l)**	51(28–111)	65.5 (35.3–172)	44 (22–79.5)	0.423
**Lactate Dehydrogenase (U/l)**	247 (128–420)	347 (204.8–607.2)	200 (87–377)	0.447
**Alkaline Phosphatase (U/l)**	226 (142–421)	295 (218.8)	184 (126–328)	0.550
**Leucocytes (g/l)**	6.7 (4.7–8.9)	7.7 (5.3–12.9)	5.8 (4.6–7.7)	0.170
**Bilirubin (mg/dl)**	1.4 (0.7–3.3)	2.8 (1.7–5.3)	1.02 (0.6–2.1)	0.375
**Total number of ERCs**	**99**	**34 (100%)**	**65 (100%)**	
**Suspected bile duct stenosis**	86	32 (94.1%)	54 (83.1%)	0.122
**Suspected bile duct stricture after OLT**	41	32 (94.1%)	9 (13.8%)	**0.029**
**Malignant disease causing stenosis**	18	7 (20.6%)	11 (16.9%)	0.653
**Suspected choledocholithiasis**	11	7 (20.6%)	4 (6.2%)	**0.030**
**Stent overall**	53	19 (55.9%)	34 (52.3%)	0.735
**plastic stent**	40	16 (47.1%)	24 (36.9%)	0.329
**metal stent**	6	2 (5.9%)	4 (6.2%)	0.957
**Metal stent recanalization**	7	2 (12.5%)	5 (15.5%)	0.832
**Received antibiotic therapy Pre ERC**	40	21 (61.8%)	19 (29.2%)	**0.002**
**Received antibiotic therapy post ERC**	94	34 (100%)	60 (92.3%)	0.097
**Overlapping microbial analysis (oral cavity/biliary system)**	33	14 (41.2%)	19 (29.2%)	0.265
**Overlapping microbial analysis (pre ERC duodenoscope/biliary system)**	12	4 (11.8%)	8 (12.3%)	0.841
**Overlapping microbial analysis (oral cavity/pre ERC duodenoscope/biliary system)**	7	3(8.8%)	4 (6.1%)	0.688
**Overlapping microbial analysis (biliary system/post ERC duodenoscope)**	78	27 (79.4%)	51 (79.6%)	0.431
**Overlapping microbial analysis (oral cavity/pre ERC duodenoscope /biliary system/post ERC duodenoscope)**	3	1 (2.9%)	2(3.1%)	0.999

**Table 2:** Uni- and multivariate analysis of risk factors for post-ERC cholangitis

Variable	HR (univariate)	*p*-value	HR (multivariate)	*p*-value
**Choledocholithiasis**	3.95 (1.07–14.64)	**0.040**	2.72 (0.63–10.02)	0.096
**Post OLT**	0.37 (0.15–0.92)	**0.032**	0.52 (0.21–1.32)	0.086
**Malignant disease**	0.18 (0.038–0.325)	0.587	–-	–-
**Smoking**	0.39 (0.22–0.57)	0.901	–-	–-
**Age**	60.61(54.75–66.47)	0.778	–-	–-
**EPT before inclusion**	0.36 (0.18–0.53)	0.627	–-	–-
**EPT during the study period**	0.66 (0.49–0.83)	0.131	–-	–-

Data are expressed as case numbers (percentage) or mean ± standard deviation, *ERC* endoscopic retrograde cholangitis, *EPT* endoscopic papillotomy, *OLT* orthotopic liver transplantation, *CRP* c-reactive protein, *mg/dl* milligrams/decilitre, *ALT* alanine transferase, *AST* aspartate aminotransferase, *U/l* Units/litre, *GGT* gamma glutamyl transferase, *g/l* grams/litre, *NAFLD* non-alcoholic fatty liver disease, *PBC* primary biliary cholangitis, *PSC* primary sclerosing cholangitis, *AIH* autoimmune hepatitis, *mg/dl* milligrams/decilitre, *G/l* grams/litre, *mmol/l* millimole/litre, *ml/min/m2* millilitres/minute/square meter, *U/l* Units/litre, *sec* second, *µg/l* microgram/litre

A suspected biliary stenosis was the most common indication for ERC (86 of 99 ERCs), including suspected non-anastomotic or anastomotic biliary strictures (NAS/AS) following liver transplantation (OLT), hepatobiliary- and pancreatic-malignancy as well as obstructive gallstone disease (Tables 1 and 2). In 53 ERCs biliary stents were placed during the intervention, of which 40 (75%) were plastic, 6 metal (11%) and in 7 ERCs plastic stents were placed within metal stents (13%). No statistically significant difference, except the level of C-reactive protein (CRP) in laboratory parameters, between patients with or without cholangitis was found (Table 1).

Interestingly, suspected biliary strictures after OLT or choledocholithiasis, as well as markedly elevated CRP levels were significantly more common in the cholangitis than in the non-cholangitis group, but after multivariate analysis adjustment, this difference did no longer reach significance (Table 2). Further analysis of multiple clinical parameters did not show a significant correlation with cholangitis in a univariate/multivariate analysis (Table 2).

Next, the correlation between microbiological test results of bile aspirates during ERC and the clinical diagnosis of cholangitis according to the Tokyo criteria was assessed. Most patients with cholangitis had a positive microbial bile test (91.2%), either with likely pathogenic or facultative pathogenic germs, whereas the first were the leading cause in cholangitis patients (76.5%). However, the same was true in the non-cholangitis group, where 86.2% had a positive microbial bile test with 81.6% likely pathogenic microbes (Table 3).

**Table 3:** Diagnostic properties of microbiological examination of bile aspirates during ERC for the diagnosis of cholangitis

	**Cholangitis**	**No Cholangitis**	**Total**
***Positive Microbiology***			
** Total**	31 (91.2%)	56 (86.2%)	**87 (87.9%)**
** Likely pathogens for cholangitis**	26 (76.5%)	53 (81.6%)	**79 (79.8%)**
** Facultative or unusual pathogens for cholangitis**	5 (14.7%)	3 (4.6%)	**8 (8.1%)**
***Negative Microbiology***	3 (8.8%)	9 (13.8%)	**12 (12.1%)**
***Total***	**34 (100%)**	**65 (100%)**	**99 (100%)**

Results are divided into negative/positive and likely pathogens and facultative or unusual pathogens/sterile (all examinations)—Numbers in parentheses indicate percentage of total cholangitis vs. no cholangitis

Any positive microbiological test had 91.2% sensitivity for the clinical and biochemical diagnosis cholangitis, whereas any negative test excluded cholangitis with a negative predictive value of 75%. In contrast, any positive microbial test in bile aspirates had a very low specificity (13.9%) for cholangitis, resulting in a positive predictive value of 35.9% in this real-life patient cohort undergoing ERC (Additional file 1). When bile cultures positive with likely pathogenic microbes (group 1) were compared to facultative pathogenic germs or sterile tests (group 2), the positive predictive value for clinical and biochemical cholangitis was 76.5% and a specificity of 60.0%. Sensitivity was 32.9% (Additional file 1).

### *Bacteroides fragilis* is more common in patients with cholangitis

**Fig. 1:**
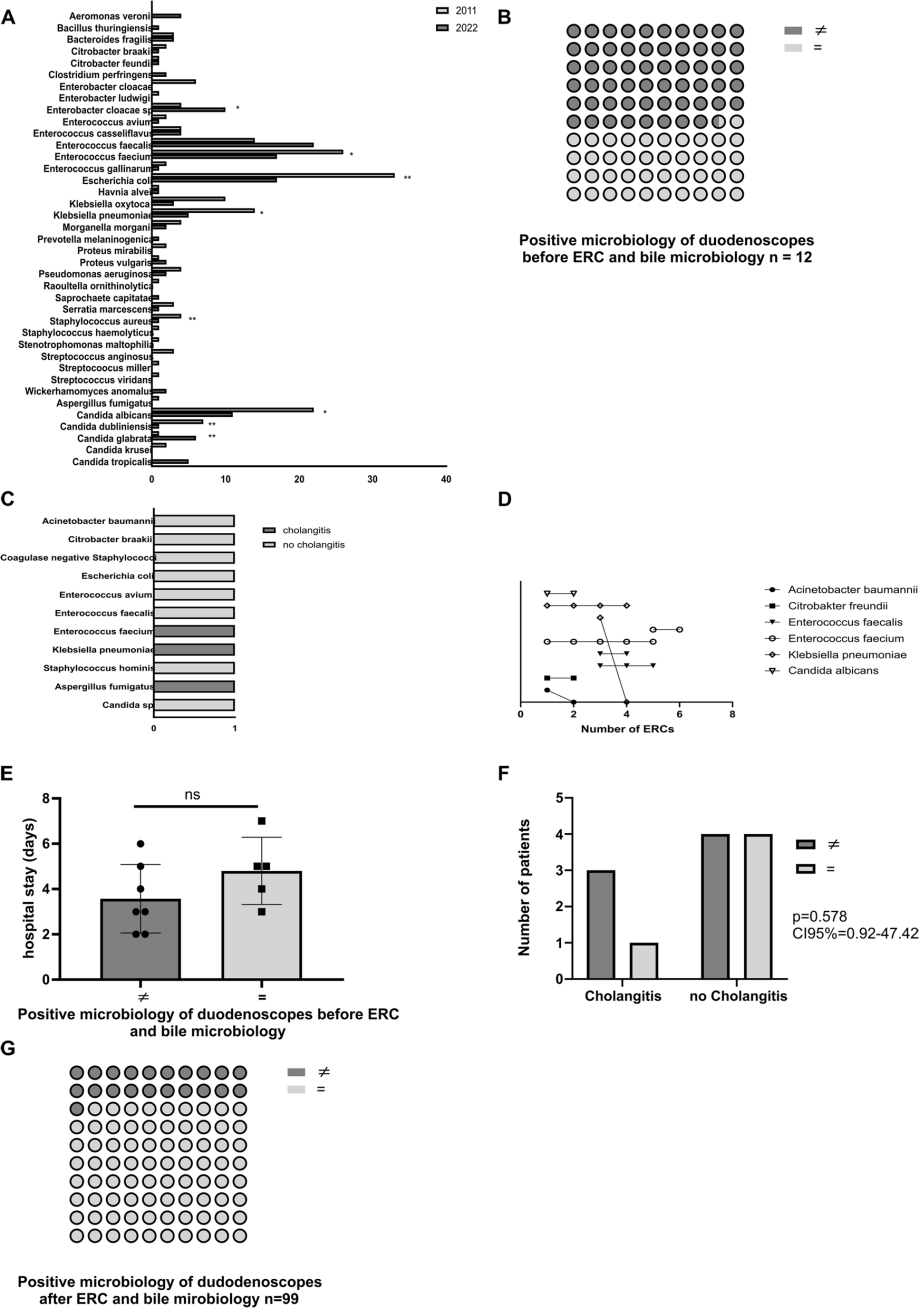
Microbial analysis of the duodenoscope and the biliary tract. Microbial bile analysis showed significant changes in the microbial composition over 10 years (**A**). Microbial analysis showed a positive result in 12 duodenoscopes before ERC (**B**) and these microbes were found in bile aspirates of these patients (**C**). A longitudinal analysis showed a high persistence rate of transmitted microbes in bile aspirates (**D**). Patients undergoing ERC with a microbial positive tested duodenoscope had no longer hospital stay (**E**) or higher cholangitis rates (**F**). After ERC in 78.8% the microbial analysis of the biliary tract and the used duodenoscopes matched (**G**). Abbreviations in order of their appearance: ERC – endoscopic retrograde cholangiography, * - *p* ˂ 0.05, ** - *p* ˂ 0.01, ns – non significant, = - positive microbiology of duodenoscope and bile acid match, ≠ - positive microbiology of duodenoscope and bile acid did not match

To evaluate if cholangitis was associated with specific microorganisms in bile aspirates, we compared the microbiological test results of patients with or without cholangitis. No difference was found in *Gram positive* or *Gram negative* bacteria as well as in fungi, which were mostly *Candida species*. Solely, *Bacteroides fragilis* (*p*=0.015) was significantly associated with cholangitis (Table 4). The most common microorganisms isolated from bile in the present study were Enterobacteriaceae, especially* E. coli*, *Klebsiella,* and* Enterococcus species. Bacteroides fragilis* and the group of facultative or unusual pathogens were significantly more frequent in patients with cholangitis compared to the non-cholangitis group. To investigate surveillance for pathogens in bile aspirates a validation cohort of patients from 2022 was analysed. The clinical data of the validation cohort are presented in Table 5. There were distinct changes in bile microbiology notable. Samples positive for *Escherichia coli* (*p*˂0.01), *Enterococcus faecium* (*p*˂0.05), *Klebsiella pneumoniae* (*p*˂0.05), *Staphylococcus aureus* (*p*˂0.01), *Candida albicans* (p˂0.05), and *Candida dubliensis* (*p*˂0.01), decreased significantly, whereas a significant increase in *Enterobacter cloacae* (*p*˂0.05), and *Candida glabrata* (*p*˂0.01), could be detected (Fig. 1A).

**Table 4:** Results from microbiological testing of bile aspirates (all examinations)

Cultured organisms	Total	No Cholangitis	Cholangitis	*p*-value
**Gram positive bacteria**				
*Enterococcus avium*	3/(96)	1/(64)	2/(32)	0.231
*Enterococcus casseliflavus*	2/(97)	1/(64)	1(33)	0.638
*Enterococcus faecalis*	11/(88)	7/(58)	4/(30)	0.881
*Enterococcus faecium*	27/(72)	18/(47)	9/(25)	0.897
*Enterococcus gallinarum*	2/(97)	2/(63)	0/(34)	0.301
*Enterococcus sp.*	7/(92)	5/(60)	2/(32)	0.739
*Staphylococcus aureus (MRSA)*	4/(95)	4/(61)	0/(34)	0.401
*Staphylococcus haemolyticus*	1/(98)	1/(64)	0/(34)	0.467
*Streptococcus anginosus*	3/(96)	3/(62)	0/(34)	0.203
*Streptococcus milleri*	1/(98)	1/(64)	0/(34)	0.467
*Streptococcus viridans*	12/(87)	5/(60)	7(27)	0.062
**Gram negative bacteria**				
*Bacteroides fragilis*	3/(96)	0/(65)	3/(31)	**0.015**
*Citrobacter braakii*	2/(97)	2/(63)	0/(34)	0.301
*Citrobacter sp.*	1/(98)	0/(65)	1/(33)	na
*Enterobacter cloacae*	6/(93)	4/(61)	2/(32)	0.957
*Enterobacter ludwigii*	1/(98)	1/(64)	0/(34)	na
*Enterobacter sp.*	4/(95)	2/(63)	2/(32)	0.999
*Escherichia coli*	34/(65)	18/(47)	16/(18)	0.054
*Hafnia alvei*	1/(98)	1/(64)	0/(34)	na
*Klebsiella oxytoca*	7(92)	6/(59)	1/(33)	0.246
*Klebsiella pneumoniae*	12/(87)	9/(56)	3/(31)	0.467
*Klebsiella sp.*	7(92)	3/(62)	4/(30)	0.188
*Morganella morganii*	4/(95)	2/(63)	2/(32)	0.999
*Pseudomonas aeruginosa*	4/(94)	1/(64)	3/(31)	0.080
*Proteus mirabilis*	2/(97)	1/(64)	1/(33)	0.999
*Proteus vulgaris*	1/(98)	1/(64)	0/(34)	na
*Raoultella ornithinolytica*	1/(98)	1/(64)	0/(34)	na
*Serratia marcescens*	3/(96)	3/(62)	0/(34)	0.203
*Stenotrophomonas maltophilia*	1/(98)	0/(65)	0/(33)	na
**Fungi**				
*Aspergillus fumigatus*	1/(98)	1/(64)	0/(34)	na
*Candida albicans*	11/(88)	8/(57)	3/(31)	0.600
*Candida glabrata*	1/(98)	1/(64)	0/(34)	na
*Candida kruseii*	2/(97)	2/(63)	0/(34)	0.301
*Candida sp.*	9(90)	5/(60)	4/(30)	0.503

Numbers indicate the number of aspirates in which the indicated organism was cultured. Numbers in parentheses indicates negative specimen

Abbreviations in order of their appearance: *ERC* endoscopic retrograde cholangiography, *na* not applicable, *MRSA multiresistent Staphylococcus aureus*, *sp* species

**Table 5:** Patients´ characteristics and characteristics of ERCs of the validation cohort

	**Total**	**Cholangitis**	**No Cholangitis**	***p*-value**
**Number of patients (%)**	51 (100%)	19 (37.3%)	32 (62.7%)	0.812
**Number of female patients**	12 (23.5%)	5 (41.6%)	7 (58.3%)	0.564
**Age (years)**	62.1 ± 13.7	63.4 ± 13.9	61.8 ± 9.2	0.279
**Smoker**	19 (37.2%)	7 (36.8%)	12 (63.2%)	0.748
**EPT before inclusion**	22 (43.1%)	9 (40.9%)	13 (36.4%)	0.391
**EPT during the study period**	30 (58.8%)	8 (26.7%)	22 (73.3%)	0.773
**Diabetes mellitus**	6 (11.8%)	3 (50%)	3 (50%)	0.522
**Malignant disease**	12 (23.8%)	5 (41.6%)	7 (58.3%)	0.684
**Malignant disease causing bile duct stenosis**	6 (11.8%)	2 (33.3%)	4 (66.6%)	0.112
**OLT prior to study inclusion**	19 (37.2%)	5 (26.3%)	14 (73.7%)	0.09
**CRP (mg/dl)**	2.1 (0.4–12.3)	6.9 (3.7–11.3)	0.8 (0.8–1.2)	**0.01**
**AST (U/l)**	64 (41–108)	71 (44.5–134.6)	55 (38–79.4)	0.201
**ALT (U/l)**	54(29–119)	73 (38–186)	34 (19–74.4)	0.399
**Lactate Dehydrogenase (U/l)**	252 (131–615)	356 (212–531)	199 (56–411)	0.399
**Alkaline Phosphatase (U/l)**	278 (99–565)	280 (110–356)	190(120–429)	0.513
**Leucocytes (g/l)**	7.5 (3.2–14.2)	7.7 (3.1–14.0)	4.6 (3.3–7.7)	0.090
**Bilirubin (mg/dl)**	1.8 (0.7–6.3)	2.2 (0.8–5.3)	0.9 (0.7–2.1)	0.299
**Total number of ERCs**	**99**	**37 (100%)**	**62 (100%)**	
**Suspected bile duct stenosis**	81	29 (78.4%)	52 (83.8%)	0.178
**Suspected bile duct stricture after OLT**	43	33 (89.2%)	10 (16.1%)	**0.032**
**Malignant disease causing stenosis**	16	6 (16.2%)	10 (16.1%)	0.712
**Suspected choledocholithiasis**	13	9 (24.3%)	4 (6.5%)	**0.025**
**Stent overall**	55	20 (54.1%)	35 (56.5%)	0.826
**Plastic stent**	43	18 (48.6%)	25 (45.1%)	0.852
**Metal stent**	4	2 (5.4%)	2 (3.2%)	0.787
**Metal stent recanalization**	3	2 (5.4%)	1 (1.6%)	0.573
**Received antibiotic therapy within 24 h pre ERC**	42	22 (59.5%)	9 (14.6%)	**0.03**
**Received antibiotic therapy post ERC**	92	37 (100%)	56 (90.3%)	0.231

Data are expressed as case numbers (percentage) or mean ± standard deviation., *EPT* endoscopic papillotomy, *OLT* orthotopic liver transplantation, *CRP* c-reactive protein, *mg/dl* milligrams/deciliter, *ALT* alanine transferase, *AST* aspartate aminotransferase, *U/l* Units/liter, *GGT* gamma glutamyl transferase, *g/l* grams/liter, *ERC* endoscopic retrograde cholangiography, *NAFLD* non-alcoholic fatty liver disease, *PBC* primary biliary cholangitis, *PSC* primary sclerosing cholangitis, *AIH* autoimmune hepatitis, *mg/dl* milligrams/deciliter, *G/l* grams/liter, *mmol/l* millimole/liter, *ml/min/m2* milliliters/minute/square meter, *U/l* Units/liter, *sec* second, *µg/l* microgram/liter

### Bile microbiology does match microbial testing of duodenoscopes before ERC

To investigate potential microbial transmission due to contaminated endoscopes, we analyzed irrigation liquid of the endoscopes before ERC. In 12 samples microbes were found in endoscopes (Table 6) and in almost one half (41.7%) of the ERCs with these endoscopes the same microbes were present in the bile of these patients (Fig. 1B). Interestingly, microbes that were found in the irrigation fluid pre ERC always resulted in a positive bile culture for the specific germ (Fig. 1C). Furthermore, in a longitudinal analysis all bacteria (except *Acinteobacter baumannii* and *Klebsiella pneumonia*) in one of three cases were present in follow-up ERCs (Fig. 1D). As expected, the microbial analysis of duodenoscopes’ irrigation liquid after ERC (Table 5) did match the microbial bile analysis of these patients in 78.8% of cases (Fig. 1G). But transmission from the duodenoscope to the biliary tract did not result in longer hospital stays (Fig. 1E) or more frequent post ERC cholangitis (Fig. 1F).

**Table 6:** Results from microbiological testing of duodenoscopes and the oral cavity

Cultured organism	Total	No cholangitis	Cholangitis	*p*-value
**Oral microbiology**				
Overall	126	81	45	
*Enterobacter cloacae*	3 (2.3%)	3 (3.7%)	0 (na)	na
*Enterobacter sp*	2 (1.5%)	2 (2.4%)	0 (na)	na
*Escherichia coli*	11 (8.7%)	10 (12.3%)	1 (2.2%)	0.991
*Enterococcus faecium*	6 (4.7%)	4 (4.9%)	2 (4.4%)	0.567
*Enterococcus sp*	5 (3.9%)	4 (4.9%)	1 (2.2%)	0.206
*Klebsiella pneumoniae*	18 (14.2%)	8 (9.8%)	10 (22.2%)	0.518
*Klebsiella sp*	1 (0.8%)	0 (na)	1 (2.2%)	na
*Proteus mirabilis*	9 (7.1%)	5 (6.1%)	4 (8.8%)	0.991
*Pseudomonas aeruginosa*	13 (10.3%)	6 (7.4%)	7 (15.5%)	0.991
*Rothia mucilaginosa*	3 (2.3%)	3 (3.7%)	0 (na)	na
*Staphylococcus aures (MRSA)*	7 (5.5%)	3 (3.7%)	4 (8.8%)	0.635
*Stenotrophomonas maltophilia*	6 (4.7%)	4 (4.9%)	2 (4.4%)	0.429
**Fungi**				
*Candida albicans*	18 (14.2%)	14 (17.2%)	4 (8.9%)	**0.002**
*Candida glabrata*	2 (1.5%)	0 (na)	2 (4.4%)	na
*Candida krusei*	2 (1.5%)	2 (2.4%)	0 (na)	na
*Candida sp*	17 (0.7%)	12 (14.8%)	5 (11.1%)	**0.038**
*Candida tropicalis*	3 (2.3%)	1 (1.2%)	2 (4.4%)	0.991
**Microbiology of duodenoscopes before ERC**				
Overall	12	9	3	
*Acinetobacter baumanii*	1(8.3%)	1 (11.1%)	0 (na)	na
*Citrobacter braakii*	1(8.3%)	1 (11.1%)	0 (na)	na
*Coagulase negative Staphylococci*	1(8.3%)	1 (11.1%)	0 (na)	na
*Escherichia coli*	1(8.3%)	1 (11.1%)	0 (na)	na
*Enterococcus avium*	1(8.3%)	1 (11.1%)	0 (na)	na
*Enterococcus faecalis*	1(8.3%)	1 (11.1%)	0 (na)	na
*Enterococcus faecium*	1(8.3%)	0 (na)	1 (33.3%)	na
*Klebsiella pneumoniae*	1(8.3%)	0	1 (33.3%)	na
*Staphylococcus hominis*	1(8.3%)	1 (11.1%)	0 (na)	na
*Staphylococcus warneri*	1(8.3%)	1 (11.1%)	0 (na)	na
**Fungi**				
*Aspergillus fumigatus*	1(8.3%)	0 (na)	1 (33.3%)	na
*Candida sp*	1(8.3%)	1 (11.1%)	0 (na)	na
**Microbiology of duodenoscopes after ERC**				
Overall	149	93	56	
*Acinetobacter pitti*	1 (0.6%)	1 (1.1%)	0 (na)	na
*Citrobacter braakii*	2 (1.2%)	1 (1.1%)	1 (1.7%)	0.999
*Coagulase negative Staphylococcus*	2 (1.2%)	2 (2.3%)	0 (na)	0.486
*Enterobacter cloacae*	6 (4%)	4 (4.3%)	2 (3.5%)	0.567
*Enterobacter ludwigii*	1 (0.6%)	1 (1.1%)	0 (na)	na
*Enterobacter sp*	7 (4.6%)	6 (6.4%)	1 (1.7%)	**0.029**
*Enterococcus avium*	3 (2%)	2 (2.1%)	1 (1.7%)	0.991
*Enterococcus casseli flavus*	2 (1.2%)	1 (1.1%)	1 (1.7%)	0.999
*Enterococcus faecalis*	10 (6.7%)	6 (6.4%)	4 (7.1%)	0.656
*Enterococcus faecium*	11 (7.3%)	10 (10.7%)	1 (1.7%)	**0.0003**
*Enterococcus gallinarum*	1 (0.6%)	0 (na)	1 (1.7%)	na
*Enterococcus sp*	4 (2.6%)	2 (2.1%)	2 (3.5%)	0.999
*Escherichia coli*	25 (16.8%)	11 (11.8%)	14 (25%)	0.572
*Elizabethkingia miricola*	1 (0.6%)	1 (1.1%)	0 (na)	na
*Klebsiella oxytoca*	7 (4.6%)	3 (3.2%)	4 (7.1%)	0.991
*Klebsiella pneumoniae*	13 (8%)	10 (10.7%)	3 (5.3%)	**0.017**
*Klebsiella sp*	6 (4%)	4 (4.3%)	2 (3.5%)	0.567
*Morganella morganii*	1 (0.6%)	1 (1.1%)	0 (na)	na
*Neisseria spp*	2 (1.2%)	1 (1.1%)	1 (1.7%)	na
*Proteus mirabilis*	1 (0.6%)	1 (1.1%)	0 (na)	na
*Pseudomonas aeruginosa*	5 (3.3%)	4 (4.3%)	1 (1.7%)	0.066
*Raoultella ornithinolytica*	1 (0.6%)	0 (na)	1 (1.7%)	na
*Serratia marcescens*	2 (1.2%)	1 (1.1%)	1 (1.7%)	0.999
*Serratia odorifera*	1 (0.6%)	1 (1.1%)	0 (na)	na
*Staphylococcus aureus (MRSA)*	5 (3.3%)	4 (4.3%)	1 (1.7%)	**0.048**
*Staphylococcus haemolyticus*	1 (0.6%)	1 (1.1%)	0 (na)	na
*Stenotrophomonas maltophilia*	2 (1.2%)	1 (1.1%)	1 (1.7%)	0.999
*Streptococcus anginosus*	1 (0.6%)	0 (na)	1 (1.7%)	na
*Streptococcus peroris*	1 (0.6%)	0 (na)	1 (1.7%)	na
**Fungi**				
*Candida albicans*	8 (5.3%)	5 (5.3%)	3 (5.3%)	0.619
*Candida glabrata*	1 (0.6%)	0 (na)	1 (1.7%)	na
*Candida krusei*	1 (0.6%)	0 (na)	1 (1.7%)	na
*Candida sp*	12 (8.1%)	7 (7.5%)	5 (8.9%)	0.684
*Candida tropicalis*	2 (1.2%)	1 (1.1%)	1 (1.7%)	0.999

Numbers indicate the number of aspirates in which the indicated organism was cultured. Numbers in parentheses indicates negative specimen

Abbreviations in order of their appearance: *ERC* endoscopic retrograde cholangiography, *na* not applicable, *MRSA multiresistent Staphylococcus aureus*, *sp* species

### Microbial transmission from the oral cavity into the biliary system

**Fig. 2:**
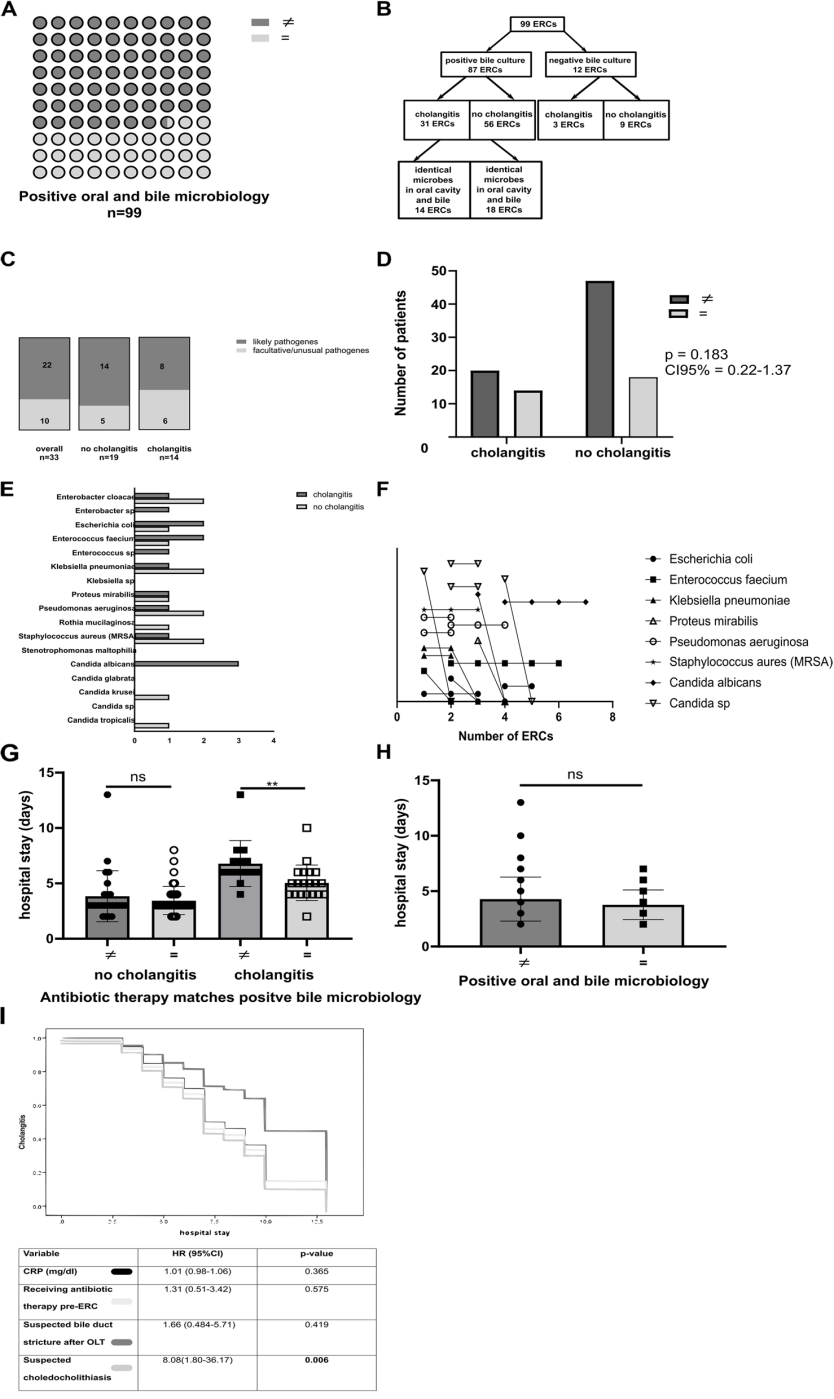
Microbial analysis of the oral cavity and the biliary tract. Microbial analysis showed identical microbial species in 33% ERCs (**A**). Identical microbial analysis in cholangitis and non-cholangitis patients (**B**). Identical microbes found in throat and bile divided into facultative or unusual pathogens and likely pathogenic microbes (**C**). Patients undergoing ERC with identical microbial analysis in the oral cavity and bile had no higher cholangitis rates (**D**). Patients with or without cholangitis had no difference in microbes transmitted to the biliary system (**E**). In a longitudinal analysis most of these microbes were persistently present in the biliary system (**F**). Whereas patients with cholangitis and antibiotic therapy matching bile microbiology had a shorter hospital stay (**G**), the same microbial analysis in the throat and bile did not lead to longer hospital stay (**H**). Multivariate Cox-Regression analysis identified solely suspected choledocholithiasis as an independent risk factor (**I**). Abbreviations in order of their appearance: ERC – endoscopic retrograde cholangiography, ns – non significant. =—positive match, ≠—negative match, CRP – c-reactive protein, mg/dl – milligrams/deciliter, HR – hazard ratio, CI – confidence interval

We analyzed the oral microbiology of these patients and mostly found fungi in the oral cavity of these 49 patients (Table 6). Candida species were barely significant more often present in the oral microbiota of patient without cholangitis. The finding that identical microbial species are present in throat and in bile samples of the same ERC in 33% of all cases suggests a translocation of oral microbiota to the bile (Fig. 2A). Of the 56 ERCs in patients without cholangitis, but with positive bile cultures, 18 cases presented with identical microbes in throat and bile; - 14 microbes of which are commonly considered as pathogenic if isolated from bile cultures (Fig. 2B)*.* On the other hand, in 14 cholangitis patients identical microbes in throat and bile were found; - 6 microbes of them are considered as non-pathogenic bacteria (Fig. 2C). The bacteria transmitted from the oral cavity to the biliary system are represented in Fig. 2E. Interestingly, *Candida albicans* was solely transmitted in patients with cholangitis, whereas *Candida krusei* and *Candida tropicalis* were only found in patients without cholangitis (Fig. 2E). Except* Candida sp* most oral pathogens transmitted to the biliary system persisted in a longitudinal analysis of 7 patients (Fig. 2F). A microbial-adapted or -matching antibiotic therapy resulted in a shorter hospital stay in this group (Fig. 2G). Interestingly, in the non-cholangitis group the rate of identical oral and bile microbes was even higher (45%), but did not result in higher cholangitis rates (Fig. 2D) and the supposed transmission from the oral cavity to the biliary tract did not lead to longer hospital stays (Fig. 2H).

Among all clinical parameters and overlapping microbial signatures in the two compartments analyzed and the pre ERC/post ERC duodenoscopes, four clinical features could be identified as relevant for cholangitis (Tables 1 and 2). These features stayed significant in a univariate Cox analysis (Table 7). Out of these four factors, only suspected choledocholithiasis remained significant in the multivariate regression model for length of hospital stay (Fig. 2I).

**Table 7:** Univariate analysis for hospital stay in patients with cholangitis

Variable	HR (univariate)	*p*-value
**CRP (mg/dl)**	7.69 (5.51–9.79)	**0.003**
**Receiving antibiotic therapy pre-ERC**	0.325 (0.127–0.532)	**0.0015**
**Suspected bile duct stricture after OLT**	0.802 (0.670–0.9351)	**0.01**
**Suspected choledocholithiasis**	0.144(0.014–0.275)	**0.03**

*ERC* endoscopic retrograde cholangiography, *CRP* c-reactive protein, *OLT* orthotopic liver transplant, *mg/dl* milligrams/deciliter

## Discussion

The microbiota and the microbiology of the bile is gaining more and more attention in terms of biliary diseases. ERC is an invasive method and although the endoscopes are reprocessed according to U.S. guidelines and manufacturers’ recommendations for cleaning and HLD process, microbes could still be transferred into the bile, since the device has multiple contacts to germ bearing surfaces during the procedure. And the endoscope might by a biohazard by itself in case of incomplete reprocessing. The key questions of our study were: (1) is there contamination of the bile during ERC, (2) where does it come from, and finally, (3) is it clinically relevant? In our study, we analyzed the oral microbiology, duodenoscopes before and after ERC, and bile from 99 procedures. We could show that the bile is regularly contaminated during ERC by pathogens either from contaminated duodenoscopes or from oral pathogen transfer.

In a recent study in patients with suspected cholangitis 91.8% had positive bile cultures with *Enterococcus species* (67.6%), *Klebsiella spp.* (44.5%), *E. coli* (40.6%), *Pseudomonas spp.* 52 (7.8%), and anaerobes (9.6%) [13]. Consistent with this report, the most common microorganisms isolated from bile in our study were Enterobacteriaceae, especially* E. coli*, *Klebsiella species,* and* Enterococcus species.* These microorganisms are the most frequent cause of cholangitis [14–16]. However, in our cohort, we found these bacteria also in bile samples from the non-cholangitis group and there was a lack of association between the presence of these bacteria and clinical or biochemical cholangitis. The finding that *Bacteroides fragilis* was the only bacteria that was significantly more frequent in patients with cholangitis supports validity of our data, since *Bacteroides fragilis* is well known to play a role in biliary infection, especially in elderly patients and patients with previous biliary surgery [15]. The metagenomic analysis has proven invasion and colonization of oral commensals in the gut of patients with cirrhosis [17]. Going along with another study showing the enrichment of oral microbes in the gut of cirrhotic patients with alcohol dependency [18], suggesting that the oral microbiome plays a key role in different liver diseases including biliary obstruction, as shown in an experimental mouse model [19]. In contrast to a previous study [20], biliary candidiasis was associated with positive fungal cultures of buccal smears in our study.

Preventing bile contamination by duodenoscopes is of utmost importance. Recent studies investigated the use of disposable duodenoscopes to prevent biliary infectious complications [21–26]. Even when the number of biliary infections could be reduced by excluding microorganisms that cannot be removed during reprocessing the duodenoscope, at least 3.8% of patients that underwent ERC with a disposable duodenoscope presented with microbial contamination, most likely, and in accordance with our data, due to the oral microbiome [27].

Independent risk factors for post-ERC cholangitis like hilar obstruction, age ≥ 60 years, and a history of previous ERC, were evaluated in two studies [28, 29]. Incomplete biliary drainage and factors causing that, like primary sclerosing cholangitis and hilar obstruction, are the main risk factor for post-ERCP cholangitis [30–33]. In our study we find certain suspected choledocholithiasis, which suggest impeded biliary drainage, as an independent risk factor for the length of hospital stay. These data underline the importance of an unhampered biliary drainage.

In several studies, assessing the contamination rate after duodenoscope reprocessing using either DHLD or ethylene oxid (EtO) sterilization, the reported contamination rate was 9.2%±0.025% [34, 35]. This is in accordance with our findings. While contamination rates of reprocessed duodenoscopes seems to be very high, our data show that microbial translocation from duodenoscopes to bile does not result in longer hospital stays or worse outcome. Microbial analysis of bile compared to analysis from used, non-reprocessed endoscopes after ERC, were highly congruent (78.8%). These facts suggest that duodenoscopes do contaminate the biliary tree but it does not affect clinical outcome.

To date, diverse gastrointestinal diseases were associated with the oral microbiome in a fairly large amount of studies [36]. The bile microbiome and the bacterial composition of the salvia demonstrate a high correlation and a relatively high similarity between the bile microbiome and duodenal microbiota were identified [37, 38]. 13 novel biliary bacteria based on whole-metagenome shotgun sequencing were identified by Shen et al. and 8 of the 13 novel species were human oral microbial taxa [39]. The microbiome of the biliary system and the upper gastrointestinal tract can be modulated by the oral microbiota directly or indirectly [40]. Oral bacteria participate in the pathogenesis of gallstone diseases [40], although a clear understanding of the mechanisms of their influence on the cholelithogenesis is lacking [41]. In patients with gall stone disease the most common inhabitants of the digestive tract are *Proteobacteria, Firmicutes, Bacteroidetes, Actinobacteria, Fusobacteria*, as well as *Synergistetes* and TM7 [42]. Interestingly *Enterococci* genera are regularly found in the oral microbiome leading to oral disease e.g. caries or endodontic infections [43]. Two *Enterobacteriaceae* genera (*E. coli*, *Klebsiella spp.*) and *Enterococcus faecium* were detected in the majority of bile samples and *Bacteroides fragili*s, belonging to the *Bacteroidetes* phyla, was associated with cholangitis in our study. Furthermore, Enberobacteriaceae genera were abundant in the oral cavity as well as in the gut microbiome of patients with colitis, suggesting that the biliary tree might also be contaminated from the oral cavity [44]. Our data are supportive for this concept: identical microbial species were present in throat and in bile samples of the same ERC in 33% of all cases. Although this pathogen transfer did not lead to more frequent cholangitis or worse clinical outcome, we assumed that the microbial analysis of bile aspirates might influence the post-ERC treatment and the duration of the hospital stay. An antibiotic treatment adapted to the microbial analysis of bile aspirates shortened length of hospital stay.

Important limitations of this study are the small number of patients and the heterogenic patient population. It may be another potential limitation, that there is no reference method for biliary sampling in our setting (e.g., via percutaneous transhepatic biliary drainage). Furthermore, our findings from this analysis of real-life data are not universally applicable but reflect treatment with mainly interventional ERC of contemporary patient populations at a tertiary referral center.

## Conclusions

In conclusion, this retrospective study shows that contamination of the bile with the oral microbiome via ERC is likely, but mostly harmless. Aspiration and microbiological sampling of bile is feasible, but interpretation of the result remains challenging. Further, more powerful studies are needed.

## Supplementary Information

**Additional file 1:** **Supplementary Table 1.**

## Data Availability

The datasets used and/or analyzed during the current study are available from the corresponding author on reasonable request.

## Abbreviations

AS: Anastomotic biliary stricture
BBE: Bacteroides Bile Esculin
CRP: C-reactive protein
DBS: Distal biliary strictures
DHLD: Double-reprocessing High-Level Disinfection
ERC: Endoscopic retrograde cholangiography
EPT: Endoscopic papillotomy
HLD: High-Level Disinfection
NAS: Non-anastomotic biliary stricture
OLT: Orthothopic liver transplant
