# Supplementary material for: Transmission of oral microbiota to the biliary tract during endoscopic retrograde cholangiography
Source: BMC Gastroenterol. 2023 Apr 3;23:103. doi: 10.1186/s12876-023-02721-7 (PMC10069052; doi:10.1186/s12876-023-02721-7)
Supplement: Supplementary file 1 — Additional file 1: Supplementary Table 1. [file 12876_2023_2721_MOESM1_ESM.docx]

**Supplementary Table 1**

|  | Microbiology  positive vs. negative | | Group 1 vs group 2 | |
| --- | --- | --- | --- | --- |
| **Sensitivity** | 91.2% | 76.32% to 98.14% | 32.91% | 22.75% to 44.40% |
| **Specificity** | 13.9 % | 6.53% to 24.66% | 60.00 % | 36.05% to 80.88% |
| **Positive likelihood ratio** | 1.06 | 0.92 to 1.22 | 0.82 | 0.44 to 1.53 |
| **Negative likelihood ratio** | 0.64 | 0.18 to 2.20 | 1.12 | 0.76 to 1.65 |
| **Disease prevalence** | 34.3% | 25.09% to 44.56% | 79.80% | 70.54% to 87.20% |
| **Positive Predictive Value** | 35.6% | 32.43% to 38.97% | 76.47% | 63.56% to 85.83% |
| **Negative Predictive Value** | 75 % | 46.49% to 91.19% | 18.46 % | 13.29% to 25.06% |
| **Accuracy** | 40.4% | 28.78% to 48.70% | 38.38% | 30.66% to 50.74% |

Results are divided into negative/positive and likely pathogens and facultative or unusual pathogens/sterile (all examinations) - Numbers in parentheses indicate percentage of total cholangitis vs. no cholangitis

Group 1 - likely pathogenic microbes, Group 2 - facultative pathogenic germs or sterile tests
